# Seminal plasma protein profiles based on molecular weight as biomarkers of sperm fertility in horned and polled Bali bulls

**DOI:** 10.14202/vetworld.2025.122-132

**Published:** 2025-01-22

**Authors:** Rasyidah Mappanganro, Herry Sonjaya, Sudirman Baco, Hasbi Hasbi, Sri Gustina

**Affiliations:** 1 Department of Animal Science, Faculty of Science and Technology, Universitas Islam Negeri Alauddin Makassar, Indonesia; 2 Animal Science Study Program, Faculty of Animal Science, Hasanuddin University, Makassar, Indonesia; 3 Department of Animal Production, Faculty of Animal Science, Hasanuddin University, Makassar, Indonesia

**Keywords:** Bali bulls, molecular weight, seminal plasma proteins, sodium dodecyl sulfate-polyacrylamide gel electrophoresis, sperm fertility

## Abstract

**Background and Aim:**

Seminal plasma proteins (SPPs) significantly influence sperm quality, playing a critical role in fertility. This study aims to investigate the molecular weight (MW) profiles of SPPs in horned and polled Bali bulls and their correlation with sperm quality parameters.

**Materials and Methods:**

Semen samples were collected from six Bali bulls (3 horned, 3 polled). Sperm quality was evaluated based on motility, viability, abnormalities, intact membranes, and acrosomes. SPPs were extracted and analyzed using one-dimensional sodium dodecyl sulfate-polyacrylamide gel electrophoresis to determine protein MWs. Pearson’s correlation was used to analyze relationships between MW profiles and sperm quality metrics.

**Results:**

SPPs were identified across a MW range of 15–165 kDa, with specific proteins showing strong correlations with sperm quality. Proteins at 50 and 46 kDa positively correlated with motility (r = –0.96), viability (r = –0.99), and intact membranes (r = –0.86). Conversely, proteins at 40 kDa negatively correlated with these parameters. A 25 kDa protein displayed a positive correlation with intact acrosomes (r = –0.93) and a negative correlation with abnormalities (r = –0.99). Differences in sperm quality metrics between horned and polled bulls were observed, with polled bulls exhibiting fewer abnormalities.

**Conclusion:**

This study highlights the potential of SPP MW profiles as biomarkers of sperm quality in Bali bulls. Proteins at 50, 46, and 25 kDa are promising markers for sperm motility, viability, and intact acrosomes, respectively. These findings could inform bull selection and reproductive management strategies. Further research is recommended to validate these biomarkers using advanced proteomic approaches.

## INTRODUCTION

Bali bulls (*Bos javanicus*) represent a significant genetic resource in Indonesia, yet current knowledge regarding their reproductive mechanisms and sperm quality remains inadequate. Detailed information on the protein profiles of seminal plasma can provide valuable insights into factors affecting sperm quality and reproductive success in bulls. Therefore, this study addresses this knowledge gap by exploring the relationship between seminal plasma protein (SPP) profiles categorized by molecular weight (MW) and sperm quality in Bali bulls. One factor that affects sperm characteristics is the composition and quality of seminal plasma, which comprises proteins that play crucial roles in fertilization. The protein content of the seminal plasma can affect the characteristics of sperm, such as motility, morphology, viability, intact membrane [1–3], and acrosome [4]. In mammals, seminal plasma is generated by the secretion of male accessory glands, which suspend sperm [5]. This fluid, called seminal plasma, is a highly complex substance containing many organic and inorganic compounds, including proteins with significant MWs. Seminal plasma primarily stimulates and supports spermatozoa by providing a nutritional and protective environment and enhancing sperm motility within the female reproductive tract [6–9]. To examine the properties and functions of proteins, it is essential to separate them. They can be isolated from other proteins or molecules based on their size, solubility, charge, and binding affinity [10, 11]. One method employed to analyze protein profiles and determine their MWs is sodium dodecyl sulfate-polyacrylamide gel electrophoresis (SDS-PAGE) [12]. These findings collectively emphasize the importance of understanding the MW of SPPs in evaluating and predicting bull semen fertility.

The study of the MW of SPPs in cattle using the one-dimensional SDS-PAGE (1D-SDS-PAGE) method has been conducted on Simmental bulls [13], Madura bulls [14], polled Bali bulls [15], and horned Bali bulls [16]. In contrast, the 2D-SDS-PAGE method was employed for Bali bulls [17]. However, research linking the MW of SPPs with sperm characteristics remains notably lacking, especially in the case of horned and polled Bali bulls. Furthermore, research on Bali bulls has demonstrated that SPP analysis based on MW can serve as a biomarker for semen quality, facilitating the selection of superior breeding bulls. Similarly, a study on Bali bulls highlighted a positive correlation between the MW of SPPs and semen quality parameters, suggesting that protein band intensity can be used as a biomarker for bull selection [16].

Research on SPPs related to sperm characteristics is essential. This study investigated the correlation between SPPs categorized by MW and sperm quality in horned and polled Bali bulls. An enhanced understanding of this domain will contribute significantly to the formulation of more effective breeding strategies, thereby improving the genetic and reproductive quality of the Bali bull population. The findings are intended to serve as additional criteria for selecting prospective breeding bulls.

## MATERIALS AND METHODS

### Ethical approval

The study was approved by the Animal Ethics Committee of the Faculty of Veterinary Medicine at Udayana University, Bali, Indonesia (number B/143/UN14.2.9/PT.01.04/2024).

### Study period and location

The study was conducted from June to August 2024 at the Integrated Service Unit for Artificial Insemination and Semen Production, Livestock, and Animal Health Service, Maros and in the *in vitro* embryo production laboratory LPPM, Hasanuddin University, Makassar, Indonesia.

### Semen collection, evaluation, and processing

Fresh semen from 42 ejaculates was collected from three-horned and three-polled Bali bulls (coded as 11777, 11457, 11745, 11442, 11540, and 11232) aged between 4 and 8 years with an average body weight ranging from 300 to 450 kg. Their feed consisted of elephant grass (10% of body weight) and 1% concentrate with 16%–17% crude protein, with water available *ad libitum*. Semen evaluations related to volume, sperm concentration, sperm motility, viability, and abnormalities concerning Hasbi *et al*. [18], and an intact membrane and acrosome, regarding Priyanto *et al*. [19].

### Determination of the MWs of SPPs

**Figure 1:**
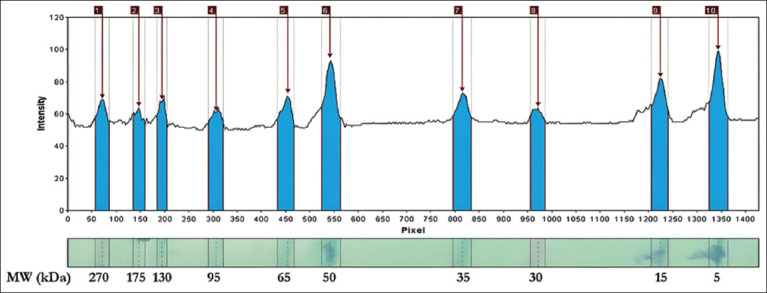
Molecular weight protein marker profile in gel analyzer 23.1.1.

**Figure 2:**
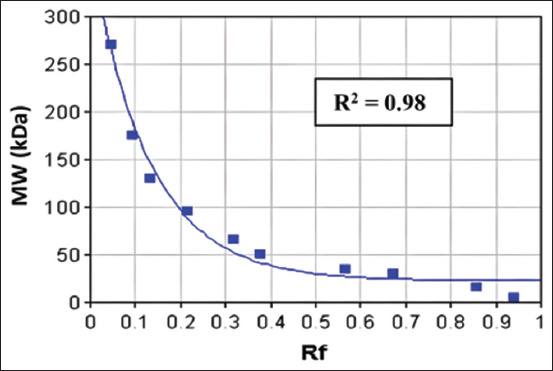
Molecular weight (kDa) calibration band of the protein standard marker with the equation of logarithm y = 342.065 * exp (7.666*x) + 22.754. Rf=Retardation factor.

The semen was centrifuged (ThermoScientific^™^, USA) at 7000× *g* for 30 min, and the supernatant was placed into straws and stored in liquid nitrogen [20]. Protein characterization was conducted using 1D SDS-PAGE based on protein MW. The protein concentration in the seminal plasma was measured using the bicinchoninic acid protein assay (ThermoScientific^™^) to analyze protein composition by molecular size. SDS-PAGE was used to detect protein bands on polyacrylamide gels. The proteins were separated using 1D SDS-PAGE (SurePAGE^™^, 4%–20% gradient gel, M00656; GenScript) and Protein Standard (Broad Multi Color Pre-Stained, M00624; GenScript), covering a MW range of approximately 5–270 kDa. Electrophoresis was performed at 200 V and 100 mA for 40 min, followed by gel staining with Coomassie Brilliant Blue (R-250; Bio-Rad, USA) for protein visualization. SDS-PAGE assessed 50 μL of lysate protein with 12% Tris-Bis polyacrylamide gels. The marker pre-stained protein ladder was loaded 4 μL into one well, and the samples were loaded in the following order (30 μL): 11777, 11457, 11745, 11442, 11540, and 11232. The MW of the protein was determined by measuring the protein bands from the acrylamide gel using the gel analyzer 23.1.1 software (Figure 1). The bands were measured to determine their MWs using the regression equation of the protein standard marker represented as the logarithmic formula *y* = 342.065*exp (−7.666 * ×) + 22.754 (Figure 2). The final MW values were calculated by converting the logarithmic Y values into their antilogarithms.

### Statistical analysis

The statistical analysis was conducted to explore the relationships between SPP profiles, characterized by MW, and sperm quality parameters in horned and polled Bali bulls. Data on sperm motility, viability, abnormalities, intact membranes, and intact acrosomes were expressed as mean ± standard deviation (SD) and analyzed for significant differences between groups using one-way analysis of variance (ANOVA). Post hoc comparisons were made using Tukey’s HSD test for pairwise group differences.Pearson’s correlation coefficient (rr) was employed to evaluate the associations between MW-specific SPPs and sperm quality parameters. Correlation strength was classified as follows: 0 ≤ r ≤ 0.30 (weak), 0.31 ≤ r ≤ 0.70 (moderate), and 0.71 ≤ r ≤ 1.00 (strong). Significance levels were set at p < 0.05 for statistical differences and p < 0.01 for highly significant differences. To determine the predictive value of specific SPP MWs on sperm quality, a heatmap visualization of correlation coefficients was generated. This provided a comparative assessment of positive and negative correlations between specific MW bands and sperm quality metrics. Additionally, descriptive statistics were used to summarize general semen quality and SPP concentrations in the two bull groups.All statistical analyses were performed using IBM SPSS Statistics version 27.0 (IBM Corp., Armonk, NY, USA). Graphical representations and data visualizations, including correlation heatmaps, were created using GraphPad Prism version 9.0 (GraphPad Software, San Diego, CA, USA). These visual tools were employed to highlight trends and patterns in the data, facilitating a clear understanding of the complex relationships between SPP profiles and sperm quality traits.

## RESULTS

### Fresh and frozen semen production

In general, fresh semen from bulls used in the study was of good quality and met the freezing requirements. The fresh semen quality of polled Bali bulls showed an average volume of 4.89 ± 2.61 mL, sperm concentration of 667.29 ± 300.70 × 10^6^ cell/mL, and straw production per ejaculate of 107.67 ± 46.82, whereas that of the horned Bali bulls showed an average volume of 4.76 ± 2.67 mL, sperm concentration of 1053.38 ± 195.38 × 10^6^ cell/mL, and straw production per ejaculate of 132.43 ± 41.00. Across all two types of bulls, there were highly significant differences (p < 0.01) in sperm concentration and frozen semen production (straw) and significantly different volumes (p < 0.05). Between the horned and polled bulls, there were highly significant differences (p < 0.01) in sperm concentration but no significant differences in volume or straw production (p > 0.05) (Table 1).

**Table 1:** Fresh semen quality of polled and horned Bali bulls and straw production.

Bull code	Fresh semen quality		Straw/ejaculates

	Volume (mL)	Sperm concentration (× 10^6^ cell/mL)	
Polled Bali bulls			
11777	2.93 ± 1.21^a^	320.29 ± 33.99^a^	52.71 ± 7.32^a^
11457	5.74 ± 2.59^b^	817.14 ± 172.32^b^	121.29 ± 34.57^b^
11745	5.98 ± 2.78^b^	864.43 ± 241.94^b,c^	149.00 ± 18.07^b^
Mean ± SD	4.89 ± 2.61	667.29 ± 300.70	107.67 ± 46.82
Horned Bali bulls			
11442	6.01 ± 2.98^b^	999.71 ± 157.79^b,c,d^	142.00 ± 29.58^a^
11540	5.60 ± 2.38^b^	1039.14 ± 246.63^c,d^	138.86 ± 41.24^b^
11232	2.65 ± 1.11^a^	1121.29 ± 180.39^d^	119.58 ± 43.72^b^
Mean ± SD	4.76 ± 2.67	1053.38 ± 195.38^b^	132.43 ± 41.00
Mean of all bulls	4.82 ± 2.60	860.33 ± 317.66	120.05 ± 45.23
Differences between bulls	*	**	**
Differences between polled and horned bulls	ns	**	ns

The different superscript within a single column indicates a difference at the 0.05 level.

* =Mean difference significant at the 0.05 level;

** =Mean difference significant at the 0.01 level. ns=Not significant, SD=Standard deviation

### Sperm quality and SPP concentration

Between individual bulls, sperm quality showed significant differences (p < 0.01) in viability and abnormality, with significant differences (p < 0.05) found in the intact membrane and acrosome. There were no differences (p > 0.05) in sperm motility or SPP concentration. The difference in sperm quality between horned and polled bulls was attributable to abnormalities at the 0.05 level. The average percentages of sperm quality of the polled Bali bulls in terms of motility, viability, abnormality, and intact membrane and acrosome were 77.14 ± 4.35, 87.35 ± 7.09, 4.96 ± 1.68, 83.00 ± 4.79, and 95.85 ± 3.00%, while those of the horned Bali bulls were 77.62 ± 4.36, 88.23 ± 7.62, 8.96 ± 7.19, 81.13 ± 3.02, and 94.80 ± 3.37%, respectively. The mean SPP concentration was 171.95 ± 0.72 μg/mL in polled bulls and 171.08 ± 0.54 μg/mL in horned bulls. The statistical analysis indicated no significant differences between the two groups (Table 2).

**Table 2:** Sperm quality and seminal plasma protein concentrations from of polled and horned Bali bulls.

Bulls code	Sperm parameters (%)					SPP conc. (μg/mL)

Motility	Viability	Abnormality	Intact membrane	Intact acrosome
Polled Bali bulls						
11777	75.00 ± 4.08	80.12 ± 5.13^a^	4.64 ± 0.81^a^	86.61 ± 9.19^b^	83.88 ± 10.73^a,b^	172.76
11457	77.86 ± 4.88	91.15 ± 4.99^b^	5.63 ± 2.15^a^	85.80 ± 5.27^b,c^	79.45 ± 3.66^b^	171.71
11745	78.57 ± 3.78	90.77 ± 4.99^b^	3.80 ± 1.14^a^	89.46 ± 2.22^a^	87.22 ± 1.95^a^	171.38
Mean ± SD	77.14 ± 4.35	87.35 ± 7.09	4.96 ± 1.68^a^	83.00 ± 4.79	95.85 ± 3.00	171.95 ± 0.72
Horned Bali bulls						
11442	78.57 ± 3.78	90.79 ± 3.16^b^	4.96 ± 1.12^a^	83.61 ± 4.71^b,d^	87.13 ± 5.16 ^a^	170.49
11540	79.29 ± 3.45	94.08 ± 2.58^b^	4.50 ± 1.79^a^	89.42 ± 2.50^a^	85.33 ± 3.46 ^a,c^	171.21
11232	77.00 ± 5.00	79.82 ± 6.85^a^	17.44 ± 6.49^a^	80.76 ± 2.20^c,d^	80.64 ± 1.05 ^b,c^	171.54
Mean ± SD	77.62 ± 4.36	88.23 ± 7.62	8.96 ± 7.19^b^	81.13 ± 3.02	94.80 ± 3.37	171.08 ± 0.54
Mean polled and horned bulls	77.38 ± 4.31	87.79 ± 7.29**	6.97 ± 5.54**	85.85 ± 5.69*	84.94 ± 5.86*	171.52 ± 0.74
Differences between individual bulls	ns	**	**	*	*	ns
Differences between polled and horned bulls	ns	ns	*	ns	ns	ns

The different superscript within a single column indicates a difference at the 0.05 level.

* =Mean difference significant at the 0.05 level;

** =Mean difference significant at the 0.01 level. ns=Not significant. SPP=Seminal plasma protein, SD=Standard deviation, conc=concentration

### Correlation between sperm quality and the number of protein bands in the seminal plasma

**Figure 3:**
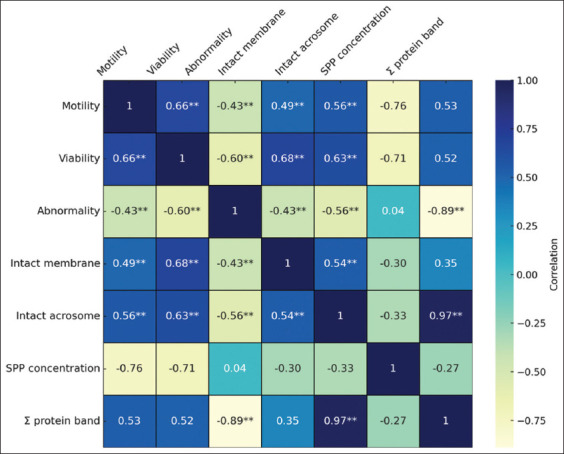
Heatmap visualization of Pearson’s correlation coefficient for sperm parameters and protein bands of seminal plasma. The scale is based on colors from blue (positive) to cream (negative); *=significant correlation p < 0.05; **=highly significant correlation p < 0.01.

Figure 3 presents the correlation coefficients between the various sperm parameters and SPP concentrations. The results of Pearson’s correlation test indicate that sperm viability has a significant positive correlation at the 0.01 level with sperm motility (0.66), intact membrane (0.71), and intact acrosome (0.63), and a negative correlation with abnormalities (−0.60). In addition, the number of protein bands showed a strong positive correlation (0.97) with intact acrosomes but a strong negative correlation with abnormalities (−0.89). Abnormality was negatively correlated with all sperm quality parameters and the number of protein bands in the seminal plasma. Table 3 presents the results of the 1D SDS-PAGE analysis, which revealed that the MW of the proteins ranged from 15 to 165 kDa. The bull with the highest number of bands was horned bull 11442, which had 16 bands, while bulls 11777, 11457, 11745, 11540, and 11232 had 14, 13, 14, 14, and 10 bands, respectively.

**Table 3:** Seminal plasma protein profile (MW) of horned and polled Bali bulls assessed by 1D-SDS-PAGE.

MW (kDa)	Bulls code						Protein presence* (%)

	11777	11457	11745	11442	11540	11232	
165	+	+	+	+	+	+	6/6 (100)
150	+	+	+	+	+	+	6/6 (100)
130	+	+	+	+	+	+	6/6 (100)
112	-	-	-	-	-	+	1/6 (16.66)
107	+	-	+	+	+	-	4/6 (66.66)
95	-	-	-	+	-	-	1/6 (16.66)
92	+	+	+	+	+	-	5/6 (83.33)
89	+	+	+	+	+	+	6/6 (100)
88	+	-	+	-	-	-	2/6 (33.33)
74	+	+	+	+	+	+	6/6 (100)
73	+	-	+	+	+	+	5/6 (83.33)
60	-	+	-	+	+	+	4/6 (66.66)
58	-	+	-	-	+	-	2/6 (33.33)
50	-	+	+	+	+	-	4/6 (66.66)
46	-	+	+	+	+	-	4/6 (66.66)
40	+	-	-	-	-	+	2/6 (33.33)
32	+	-	-	+	-	-	2/6 (33.33)
25	+	+	+	+	+	-	5/6 (83.33)
21	+	-	-	-	-	-	1/6 (16.66)
18	-	+	+	+	-	-	3/6 (50)
15	+	+	+	+	+	+	6/6 (100)
Σ Bands	14	13	14	16	14	10	

* n (%); MW (molecular weight); + (protein expressed); – (protein non-expressed). 1D-SDS-PAGE=One-dimensional sodium dodecyl sulfate-polyacrylamide gel electrophoresis

### Correlation between sperm quality and MW SPP levels

**Figure 4:**
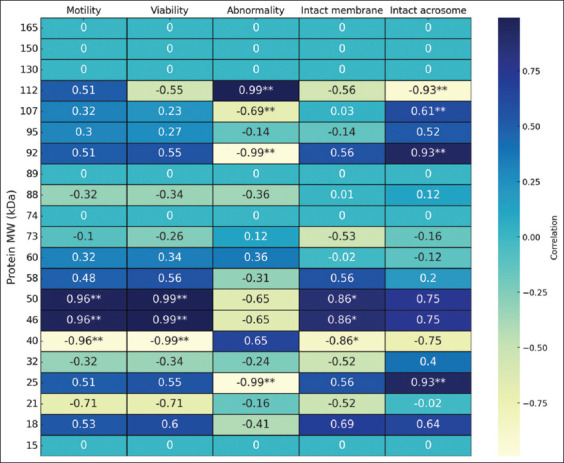
Heatmap visualization of Pearson’s correlation coefficient between the molecular weight of seminal plasma protein and sperm parameters. The scale is based on colors from blue (positive) to cream (negative). *=significant correlation p < 0.05; **=highly significant correlation p < 0.01.

The expression of protein fractions in seminal plasma showed both positive and negative correlations with various sperm parameters in Bali bulls studied (Figure 4). Proteins with MWs of 165, 150, 130, 89, 74, and 15 kDa were detected in all bulls, but their presence showed no correlation with motility, viability, abnormality, or an intact membrane or acrosome. MW of 112 kDa exhibited a strong negative correlation (−0.99) with intact acrosomes and a strong positive correlation (0.99) with abnormalities, whereas MW of 92 kDa had a strong negative correlation (–0.99) in abnormality and a strong positive correlation (0.93) with intact acrosomes. MW of 50 and 46 kDa showed strong positive correlations with motility (0.96), viability (0.99), and intact membrane (0.86), whereas 40 kDa had strong negative correlations with sperm motility (−0.96), viability (−0.99), and intact membrane (−0.86). Finally, proteins with an MW of 25 kDa displayed a significant (p < 0.01) strong negative correlation (−0.99) with abnormality but a positive strong correlation with intact acrosomes (0.93).

## DISCUSSION

The fresh semen analysis indicated that it was of good quality and met national standards; the quality intended for freezing should have a sperm motility value >70% and a sperm abnormality value <20% [21]. Previous research on the quality of fresh semen from horned Bali bulls was reported by Mappanganro [22], who found that semen volume was 4.60 ± 1.69 mL, with a concentration of 1995.29 ± 35.46 × 10^6^ cells/mL, and frozen semen production of 195.7 5 ± 85.72 straws. The semen quality of polled Bali bulls reported by Hasbi *et al*. [18] was characterized by a volume of 6.02 ± 2.22 mL and a concentration of 310 ± 12.00 × 10^6^ cells/mL, whereas Diansyah *et al*. [15] reported sperm motility of 82.91% ± 1.34% and viability of 91.97% ± 10.6%.

Sperm motility values observed in all bulls were lower than those reported by Indriastuti *et al*. [23]. In addition, individual variations in sperm motility have been documented in the fresh semen of Madura [14] and Simmental bulls [13]. Motility is another important parameter for fertilization. Sperm motility, an indicator of sperm fertility, indicates suitable spermatogenesis and maturation during epididymal transit. Mapel *et al*. [24] also reported that sperm motility was negatively correlated with abnormalities. In humans, progressive motility is significantly influenced by the oxidation of thiols in flagellar proteins, such as outer dense fiber protein 1, into disulfides during epididymal transit, facilitating sperm motility [25]. In species that receive external fertilization, sperm exposure to the fertilization medium typically results in hypotonic shock, which activates motility [26]. In mammals, progressive sperm motility is associated with cAMP signaling and adenylyl cyclase activity [27].

The higher the percentage of viable sperm, the lower the abnormality, as supported by Druet *et al*. [28] and Ducrocq and Humblot [29], who found that in Holstein and Normande bulls, viability was negatively correlated with abnormalities (−0.20 and −0.37). Sperm abnormalities may arise due to disruptions during spermatogenesis or the maturation process in the epididymis. Al-Makhzoomi *et al*. [30] noted that abnormal spermiogenesis can cause defects in chromatin condensation, leading to abnormalities in the sperm head. Issues with sperm maturation within the epididymis can result in immature sperm, which is often indicated by a proximal cytoplasmic droplet. The range of sperm abnormalities observed in this study was comparable to the percentage of total sperm abnormalities in Friesian-Holstein bulls reported by Purwantara *et al*. [31]. Furthermore, the sperm viability values obtained in this study were higher than those reported by Gustina *et al*. [32].

The intact sperm membrane is crucial for effective sperm function and fertility, as it is vital for maintaining homeostasis [33]. The results obtained for intact membranes were higher than those reported by Hasbi *et al*. [18]. Variations in the sperm’s intact membrane may be attributed to differences in the proportion of cholesterol in the sperm membrane. Sheriff and Ali [34] reported that cholesterol, an amphipathic molecule, influences intact membranes. The integrity of the sperm membrane is essential for maintaining the ability of sperm to move effectively. When the membrane is intact, it helps to maintain the appropriate structure and function of the sperm’s flagella, allowing for efficient movement toward the egg and maintaining the integrity of the acrosome, thus ensuring that the sperm’s genetic information is transmitted accurately during fertilization [35–37]. Sperm fertility relies on the integrity of the plasma membrane, which is essential for various physiological processes, including capacitation, acrosomal reaction, and zona binding [38]. Sperm with damaged membranes is more susceptible to structural defects, such as abnormally shaped heads and tails, which can disrupt their function. A high percentage of intact membranes reduce the likelihood of such abnormalities, ensuring that more sperm are morphologically normal and functional [39]. Damage to the sperm membrane can cause various abnormalities, such as malformation or deformation, thereby reducing the chances of successful fertilization.

In this study, intact acrosomes were positively correlated with intact membranes. The acrosome contains enzymes that help sperm penetrate the outer layer of the egg during fertilization [35–37]. Other studies have also found that an intact acrosome is positively correlated with live sperm, motility, and the hypo-osmotic swelling test, suggesting its link to an intact membrane in both freezable and non-freezable ejaculates [40–42] and indicating its importance in assessing sperm fertility [43]. An intact acrosome is crucial for successful fertilization, as the fusion of the outer acrosomal membrane and the overlying sperm membrane releases lytic enzymes immediately before or on contact with the zona pellucida around the oocyte [44], with the result potentially serving as a biomarker for male fertility [45].

The plasma protein concentrations determined using the Bradford method were used only as the basis for subsequent electrophoresis or mass spectrometry (MS) analyses. Therefore, plasma protein concentration alone is not a reliable indicator of semen quality [46]. However, research has shown that plasma protein components in semen effectively enhance sperm quality [47, 48]. Baharun *et al*. [49] found that SPP concentration in Simmental bulls was negatively correlated with motility, sperm concentration, and an intact acrosome, while García *et al*. [50] found that cat semen plasma cholesterol and triglyceride concentrations, along with specific protein bands, can improve the evaluation of semen quality.

Sperm quality is also based on the presence of specific proteins in the seminal plasma. The SDS-PAGE analysis revealed that the MW protein ranged from 15 to 165 kDa (Table 3). The results obtained in this study are consistent with previous research conducted by Iskandar *et al*. [16], who found levels of 15–180 kDa in horned Bali bulls; Diansyah *et al*. [15], who reported 12–150 kDa in polled Bali bulls; Azizah *et al*. [14], who registered 12–110 kDa in Madura bulls; Almadaly *et al*. [51], who found 8.5–185.5 kDa in Holstein-Friesian bulls; Almadaly *et al*. [52] who found 5.2–185.8 kDa in Japanese black bulls; Alyethodi *et al*. [53], who reported 17–245 kDa in Frieswal bulls; and Maulana *et al*. [54], who registered 11–155 kDa in Toraya buffalo. The number of protein fractions and their MWs expressed in seminal plasma may vary among species. In addition, protein molecules associated with sperm can influence their structure and metabolism, which vary among individuals [55]. Seminal plasma contains numerous proteins with specific association and dissociation relationships under certain peptide conditions based on their MWs [56].

Proteins with MWs of 165, 150, 130, 89, 74, and 15 kDa did not influence the sperm quality of Bali bulls. This finding differs from those of Karunakaran *et al*. [57], who found that heparin-binding protein with a MW of 134–101 kDa in sperm had a high correlation with the *in vitro* sperm quality of black Bengal bucks. Our findings differ from those of Almadaly *et al*. [58], who found that 11, 13, and 22.5 kDa protein bands were fertility-associated proteins in Barki rams’ seminal plasma. Cheema and Babbar [59] also found that certain SPPs, including those around 130–145 kDa, were associated with higher sperm motility, intact membranes, and better outcomes after cryopreservation. Proteins ranging in size from 14 to 205 kDa were identified in Jersey bulls, which showed significant correlations with fertility rates, emphasizing the broader importance of sperm proteins in fertility assessment [60].

MW protein of MW 112 kDa was found in the bull with the highest number of sperm abnormalities (bull code 11232). One of the causes of sperm cell abnormalities is oxidative stress, which can lead to various types of damage in sperm cells, contributing to the formation of abnormal cells. These abnormal sperm cells may result in infertility. The MW of MCTP1 is 112 kDa, which contains two transmembrane regions and three C2 domains with high calcium-binding affinity. The protein is found intracellularly and is potentially involved in the cellular response to oxidative stress [61].

A MW of 92 kDa is linked to male fertility, particularly the percentage of intact acrosomes essential for sperm-egg fusion. It releases enzymes such as hyaluronidase when it contacts the oocyte’s zona pellucida. Gelsolin (GSN), one of the most abundant actin-binding proteins, regulates the assembly and disassembly of actin filaments in cells. During sperm capacitation, the elongation of these filaments is controlled by enzymes such as phospholipase D and calcium/calmodulin-dependent protein kinase [62]. The calcium-dependent protein plays a significant role in cytoskeleton organization, cell migration, shape, and metabolism [63]. GSNs are also involved in actin dynamics, which are essential during sperm capacitation and the acrosome reaction. It facilitates the restructuring of the sperm cytoskeleton, enhancing sperm motility and interaction with the oocyte [64]. In guinea pig sperm, GSN and actin were found in the combined plasma and outer acrosomal membranes, but both proteins were absent in the membranes of capacitated spermatozoa [65].

MWs of 50 kDa and 46 kDa were strongly correlated with motility, viability, and an intact membrane. These results are in line with those of Simpson and Holmes [66]; the 46 kDa SPP, or membrane cofactor protein, is a significant component of seminal plasma associated with the plasma membrane. This protein is present in the seminal plasma of both fertile and vasectomized males, indicating that its existence is not strictly dependent on the presence of sperm.

A MW of 40 kDa suggests that this protein is required when sperm motility decreases. Sorbitol dehydrogenase (SORD) catalyzes the conversion of sorbitol into fructose within the metabolic pathway, which is crucial because sorbitol can play a role in certain pathological conditions, such as neuropathy and autosomal distal hereditary motor disorder [67, 68]. The SORD protein maintains a metabolic balance within cells and tissues. Sorbitol may serve as an alternative energy source for sperm motility [69].

A MW of 25 kDa indicates that the protein is vital for maintaining intact sperm cell acrosomes. It refers to glutathione peroxidase 6 (GPX6), which contains selenium as selenocysteine [70], which has a lower reduction potential than cysteine, making it highly suitable for incorporation into proteins that participate in antioxidant activities [71]. GPX6 is present in both sperm and seminal plasma. In sperm, GPX6 is mainly located in the mitochondrial matrix, whereas seminal GPX6 is believed to originate in the prostate [72]. In addition, GPX is expressed in the epididymal head and secreted into the semen [73]. This protein was not found in bulls with the lowest abnormalities. In line with Atig *et al*. [74], results demonstrated that GPX6 was significantly lower in humans with abnormal sperm, especially those with severe sperm pathology. The primary role of GPX6 is to protect the sperm membrane from lipid peroxidation and protect sperm DNA from oxidative damage and chromatin condensation [3].

## CONCLUSION

This study provides a comprehensive analysis of SPP profiles in horned and polled Bali bulls, highlighting their correlation with critical sperm quality parameters. Proteins at specific MWs, such as 50, 46, and 25 kDa, showed strong positive associations with sperm motility, viability, and intact acrosomes, suggesting their potential as biomarkers for fertility. Conversely, proteins at 40 kDa and 112 kDa were negatively correlated with these parameters, indicating their potential as markers of reduced sperm quality.The research offers novel insights into the role of SPP profiles in evaluating and predicting sperm quality, a critical factor for reproductive success. The use of one-dimensional sodium dodecyl sulfate-polyacrylamide gel electrophoresis (1D SDS-PAGE) to assess protein MWs and the application of robust statistical methods add rigor to the findings. By comparing horned and polled Bali bulls, this study also addresses genetic variability in fertility traits, contributing to livestock management practices. Despite its strengths, the study is limited by its reliance on 1D SDS-PAGE, which does not identify specific proteins or their functional roles. Additionally, the sample size, restricted to six bulls, may not fully represent the broader population of Bali bulls. Environmental factors and diet were controlled to some extent but may still influence sperm quality and protein expression.To build on these findings, future research should utilize advanced proteomic techniques, such as nano-liquid chromatography coupled with tandem mass spectrometry (LC-MS/MS), to identify and characterize specific fertility-related proteins. Expanding the sample size and including other cattle breeds would enhance the generalizability of the results. Furthermore, longitudinal studies examining how environmental factors and breeding interventions influence SPP profiles could provide valuable insights into optimizing reproductive outcomes. In conclusion, this study underscores the potential of SPP MW profiles as biomarkers for sperm quality in Bali bulls, offering a foundation for improved breeding strategies and genetic management. These findings contribute to the sustainable enhancement of livestock productivity and genetic quality in Bali bulls.

## AUTHORS’ CONTRIBUTIONS

RM: Data curation, investigation, methodology, writing original draft, and editing; HS: Supervision, project administration, review, and editing. SB: Investigation and methodology. HH: Conceptualization, involved in data collection, review, and editing. SG: Preparation and critical review of the manuscript and validated data. All authors have read and approved the final manuscript.
